# Dye-Sensitized Solar Cells with Modified TiO_2_ Scattering Layer Produced by Hydrothermal Method

**DOI:** 10.3390/ma18020278

**Published:** 2025-01-09

**Authors:** Yu-Shyan Lin, Wei-Hung Chen

**Affiliations:** 1 Department of Materials Science and Engineering, National Dong Hwa University, Hualien 974301, Taiwan; 2 Department of Opto-Electronic Engineering, National Dong Hwa University, Hualien 974301, Taiwan

**Keywords:** DSSC, hydrothermal, scattering, hydrangea, aggregate

## Abstract

This work proposes dye-sensitized solar cells (DSSCs) with various photoanode designs. A hydrothermal method is used to synthesize hydrangea-shaped TiO_2_ (H-TiO_2_) aggregates. The X-ray diffraction (XRD) pattern of H-TiO_2_ reveals only an anatase phase. No peaks of any other phases are detected, indicating that the hydrangea-shaped TiO_2_ is phase-pure. The size of the synthesized H-TiO_2_ is approximately 300 nm to 2 μm, and its particle size is suitable for use in the scattering layer of a DSSC. Mixing the P25 TiO_2_ into the H-TiO_2_ aggregate with the best mixing ratio can significantly improve the conversion efficiency of DSSCs. When the ratio of H-TiO_2_:P25 TiO_2_ = 3:7, the scattering layer has the optimal parameters, as determined experimentally. The optimal structure is a double layer that is formed of five layers of P25 TiO_2_ plus a single scattering layer. An open circuit voltage (*V*_oc_) of 0.77 V, short-circuit current (*J*_sc_) of 15.26 mA/cm^2^, fill factor (FF) of 0.71, conversion efficiency (*η*) of 8.33%, and charge-collection efficiency (*η*_cc_) of 0.96 are obtained from the optimally designed photoelectrode. To the best of the authors’ knowledge, this work is the first in which large particles of hydrangea are mixed with small particles of P25 TiO_2_ in various proportions to form a scattering layer.

## 1. Introduction

Global warming causes the greenhouse effect and global climate anomalies. In recent years, these issues have attracted substantial attention. The underlying causes are excessive carbon dioxide emissions and pollution. Accordingly, renewable energy is valued and is gradually being developed. The advantage of renewable energy is that it is inexhaustible. It includes solar energy, which is indispensable. Solar energy is abundant. Such advantages make solar energy a green energy that attracts much attention and has led to the establishment of an industry that is highly valued around the world [1,2].

Silicon-based solar cells dominate the commercial market at the present time. However, their manufacturing needs high vacuum equipment, which results in high manufacturing costs.

Compound solar cells can be mainly divided into II–VI and III–V series. Many material systems have been extensively and intensively studied [3,4,5,6,7]. The main materials of the II–VI compound solar cells are CdTe, CuIn_1−x_Ga_x_Se_2_ (CIGS), etc. [3,4,5]. However, due to the high toxicity of CdTe and CIGS, and the limited raw materials required, there are still concerns about environmental protection and sustainability. The main materials of the III-V compound solar cells are GaAs, GaInP, etc. [6,7]. Its conversion efficiency is very high and has been used on space satellites. However, it is difficult to manufacture on a large scale, which is also a shortcoming of this type of solar cell.

A dye-sensitized solar cell (DSSC) is a third-generation solar cell and has the following characteristics: (1) its cost is not high; (2) its manufacturing process is easy, and the cost of the required equipment is not high; (3) it can be produced with a large area; (4) it is less affected by the angle of sunlight and high-temperature environment [8]. These properties make DSSC a competent contender not only in replacing other solar cell technologies but also in building integrated photovoltaic applications.

Titanium dioxide (TiO_2_) has been successfully used in metal-oxide-semiconductor high-electron mobility transistors (MOS-HEMTs) owing to its high dielectric constant [9]. Generally, most working electrodes in DSSC are made of TiO_2_, mainly because it has the following characteristics: (1) a high specific surface area and roughness factor, (2) porosity, (3) high conductivity, (4) transparency, and (5) high chemical stability [8].

The proper use of readily available solar energy can alleviate the current problems of environmental pollution and global warming. Solar energy is not only a renewable energy but also a clean energy. The several ways to improve the efficiency of dye-sensitized solar cells target include the (1) working electrode, (2) counter electrode, (3) dye, and (4) electrolyte. Light-scattering is a well-known method for boosting the optical absorption of the photoelectrode in a DSSC [10,11,12,13,14,15,16,17,18,19,20,21,22,23,24,25,26,27,28,29]. This research is concerned with improving the photoelectric conversion efficiency of solar cells by focusing on the scattering layer in the photoanode. The manufacturing process is simple, and experimental results reveal success.

Hydrangeas MoS_2_ has been successfully synthesized and applied to the counter electrode of a DSSC [30]. Although hydrangea-shaped TiO_2_ (H-TiO_2_) has been used as a scattering layer [15], the relevant research is quite limited. In this investigation, the scattering layer in the photoanode is modified. The effects of the mixing ratio of H-TiO_2_ to P25 TiO_2_ on the characteristics of DSSCs are studied comprehensively.

Table 1 compares the performance of our studied DSSC and the previously reported DSSCs with various scattering layers [15,16,17,18,19,20,21,22,23,24,25,26,27,28,29]. The short-circuit photocurrent density (*J_sc_*), open-circuit voltage (*V_oc_*), fill factor (FF), and conversion efficiency (*η*) are listed. The conversion efficiency of our studied DSSC with the 3H7C scattering layer compares favorably to the results of other authors [15,16,17,18,19,20,21,22,23,24,25,26,27,28,29]. Our experimental results clearly demonstrate that the studied DSSC with an optimally designed photoanode is a candidate for high-performance solar cells.

## 2. Experimental

### 2.1. Preparation of Working Electrode Paste

In this experiment, commercial Degussa P25 TiO_2_ ready-made powder (80% anatase, 20% rutile) (UniRegion Bio-Tech, Hsinchu, Taiwan) was used to make the working electrode. To increase the dye adsorption capacity, high-viscosity (30–50 mPs) and low-viscosity (5–15 mPas) ethyl cellulose (Sigma Aldrich, St. Louis, MA, USA) were used to control the viscosity of the paste and increase the porosity after sintering [31]. The boiling point of ethanol is low. In order to avoid cracking during sintering due to the poor heat resistance of the film, α-terpineol (95%) (Showa Chemical Industry Co., Ltd., Tokyo, Japan) was first added as a mixing agent of the paste [31]. Then pure ethanol (>99.8%) (ECHO Chemical Co., Ltd., Miaoli, Taiwan) was added dropwise to dissolve the ethyl cellulose. Finally, a stirring hot plate (CORNING PC-420D, Corning, NY, USA) was used to stir at 650 rpm/85 °C until the ethanol had evaporated.

### 2.2. Preparation of Scattering Layer Paste

First, hydrangea-shaped TiO_2_ nano aggregates were prepared. A 40 mL volume of de-ionized (DI) water was added to the beaker. A 0.3958 g mass of ammonium hexafluorotitanate (IV) (99.99%) (Acros Organics, Geel, Belgium) was added to the DI water and stirred with a magnet for 30 min until dissolved. Then, 4.8048 g of urea (CON_2_H_4_) (99%) (Showa Chemical Industry Co., Ltd., Tokyo, Japan) was added to the above solution, with continued stirring with a magnet until it had completely dissolved. Finally, 1 mL of polyethylene glycol (PEG-600) (Shimakyu Co., Ltd., Osaka, Japan) was dropped into the solution, which was stirred for 30 min. The PEG could modify the TiO_2_ surface [32] or it prevented the TiO_2_ film from cracking when the film was dried [33].

**Figure 1:**
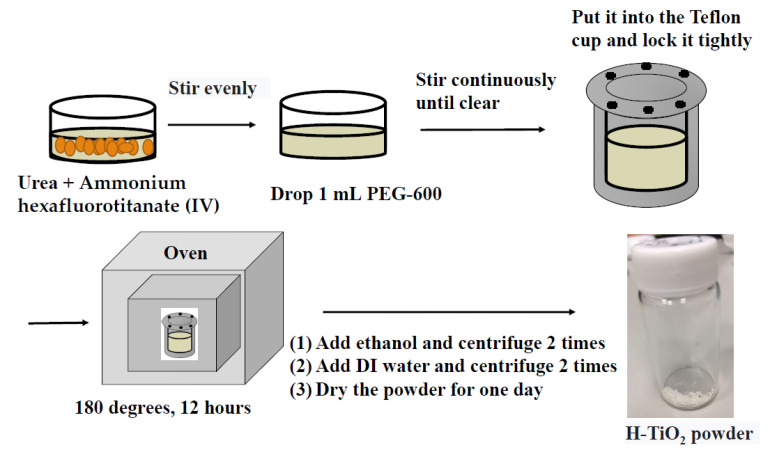
Key processing steps to obtain H-TiO_2_ powder.

The above mixture was poured into a Teflon cup and put into a hydrothermal autoclave. The accessories for this hydrothermal process were purchased from an agent (Shin, Hualien, Taiwan). The hydrothermal temperature was 180 °C, and this temperature was maintained for 12 h. After the hydrothermal reaction was completed, the mixture was cooled to room temperature. DI water and ethanol were used in the centrifugation step. This latter step was repeated twice. Finally, the mixture was put into an oven (DOS30, DENGYNG INSTRUMENTS CO., LTD, New Taipei City, Taiwan) to dry at 80 °C for 24 h. Thus, H-TiO_2_ powder was obtained. Figure 1 shows the key process steps in making H-TiO_2_ powder.

Finally, the H-TiO_2_ powder was mixed with commercial P25 TiO_2_ (C-TiO_2_) powder, as indicated in Table 2, which provides the nomenclature for scattering paste. For example, paste 3H7C contained a mixture of H-TiO_2_ and commercial P25 TiO_2_ in a ratio of 3:7. The production process of the scattering layer paste was similar to the process for preparing the working electrode paste. Ethyl cellulose was added to adjust the viscosity and increase the porosity after sintering. α-terpineol was added dropwise as a mixture, and a stirring hot plate was used to stir until the ethanol had evaporated.

### 2.3. DSSC Fabrication

To prevent corrosion by direct contact between the electrolyte and the FTO glass substrate (Ruilong Optoelectronics Co., Ltd., Miaoli, Taiwan), a dense layer of TiO_2_ nanoparticles had to be formed on the glass substrate [34]. Accordingly, for pretreatment, the substrate was soaked in a solution of 40 mM TiCl_4_ (99.9%) (Showa Chemical Industry Co., Ltd., Tokyo, Japan) in de-ionized water for 30 min at 70 °C. It was then annealed at 450 °C for 30 min. The dense layer was thus produced by hydrolysis. The hydrolysis reaction is as follows.

TiCl_4_ + 2H_2_O → TiO_2_ + 4 HCl (1)

Then, TiO_2_ paste was coated on the pretreated FTO glass by screen printing to complete the working electrode. In order to increase the adsorption of dye on the TiO_2_ thin film, a step similar to TiCl_4_ pretreatment, called TiCl_4_ post-treatment, was carried out [35,36].

N719 dye (ECHO Chemical Co., Ltd., Miaoli, Taiwan) was mixed with ethanol to form a 5 × 10^−4^ M dye solution. The solution was oscillated for 1 h using an ultrasonic cleaner (DC300, DELTA, ULTRASONIC CO., LTD., New Taipei City, Taiwan) to disperse the dye in the ethanol. Finally, the working electrode was soaked in N719 dye and put in a dark environment at 30 °C for 24 h. Acetonitrile (ACN) solution was used as the solvent in the commercial electrolyte solution (Ruilong Optoelectronics Co., Ltd., Miaoli, Taiwan). To inject the electrolyte into the DSSCs, two tiny holes had to be drilled in the FTO glass of the counter electrode. After the FTO glass had been cleaned as described in the previous steps, 3M tape was used to define the working area before it was coated with commercial platinum (Pt) paste (Ruilong Optoelectronics Co., Ltd., Miaoli, Taiwan).

DSSC assembly is the most important part of the process because of the possibility of the leakage of toxic electrolytes. The steps, over which much care must be taken, are as follows: The photoanode and Pt-coated counter-electrodes were assembled into a sealed sandwich-type cell. Efficient sealing was obtained by heating the two electrodes with hot-melt Surlyn (Ruilong Optoelectronics Co., Ltd., Miaoli, Taiwan), which served as a spacer between the electrodes. The electrolyte solution was injected through the pre-drilled holes on the counter-electrode, and the openings were sealed with a piece of glass.

## 3. Results and Discussion

### 3.1. Material and Device Analyses

The X-ray diffractometer that was used in this experiment was a Rigaku D/MAX-2500 (Rigaku, Tokyo, Japan). It was a low-angle X-ray diffractometer with a power of 18 kW. A field-emission scanning electron microscope (FE-SEM) (JEOL-7000F, Tokyo, Japan) was used to observe the morphology and surface properties of materials. A ultraviolet-visible (UV-Vis) spectrometer (Jasco V-650, Tokyo, Japan) was also used.

Photocurrent density-voltage (*J*-*V*) characteristics were measured under illumination by a simulated AM1.5G solar light from a Class AAA 550-W Xenon lamp solar simulator (ABET Technologies Sun 3000, Abet Technologies, Inc., Milford, CT, USA). Intensity-modulated photovoltage spectroscopy (IMVS) and intensity-modulated photocurrent spectroscopy (IMPS) were carried out by an electrochemical workstation (Zennium, Zahner, Germany).

#### 3.1.1. X-Ray Diffraction Analysis of the Scattering Layer

**Figure 2:**
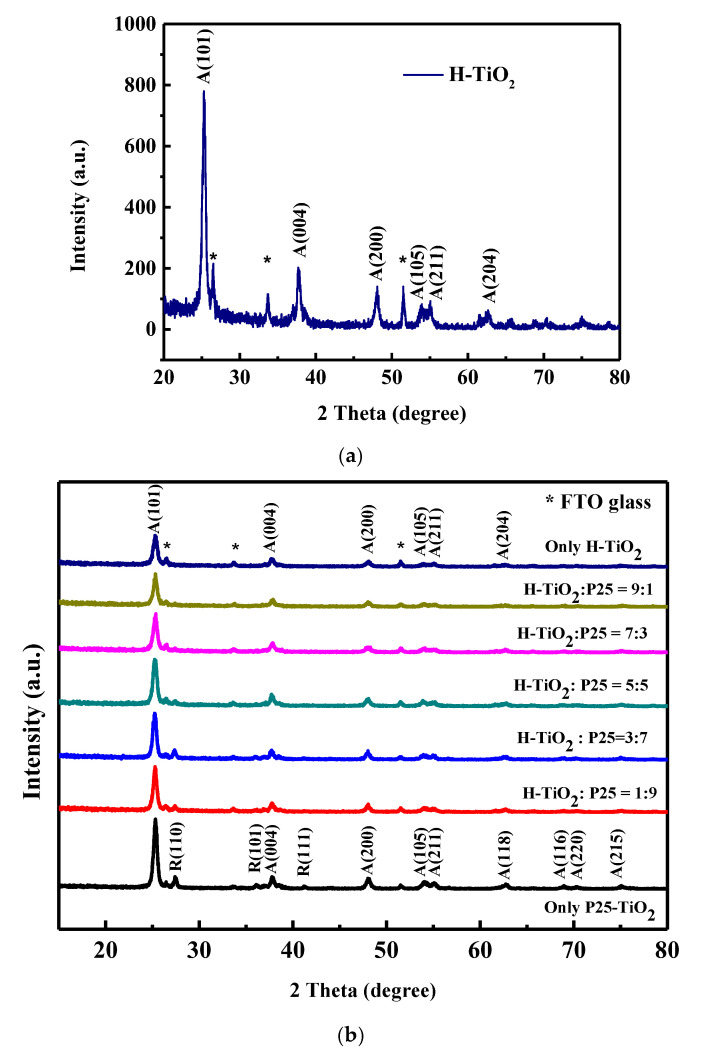
X-ray diffraction patterns of (**a**) H-TiO_2_ and (**b**) comparison of our studied films. (The * symbol represents a signal from the FTO glass substrate.)

Figure 2a displays the X-ray diffraction (XRD) patterns of the H-TiO_2_. The * symbol represents a signal from the FTO glass substrate. Jade 5.0 software is used to fit the X-ray diffraction pattern. The XRD diffraction pattern proves that the hydrangea-shaped H-TiO_2_ is an anatase phase. 2θ = 25.3°, 37.8°, 48.1°, 53.9°, 55.1°, and 62.7° correspond to the lattice planes (101), (004), (200), (105), (211), and (204) respectively. No characteristic peak of the rutile phase is obtained.

Figure 2b compares the XRD spectra for our studied films. The bottom line in Figure 2b shows the fitting result for the XRD diffraction pattern of P25 TiO_2_. P25 TiO_2_ has an obvious peak at 2θ = 25.35°, which is characteristic of the anatase phase plane (1,0,1) (JCPDS card number 21-1272). Characteristic peaks of the planes (1,1,0), (1,0,1), and (1,1,1) of the P25 TiO_2_ rutile phase are also observed, revealing that P25 TiO_2_ has anatase and rutile phases. An obvious characteristic peak near 2θ = 25.3° is the anatase phase. A larger amount of H-TiO_2_ yields a smaller (1,0,1) peak because larger hydrangea-type H-TiO_2_ particles yield a smaller peak.

#### 3.1.2. Surface Morphology of Scattering Layer

The surface morphology of the scattering layer is studied using a FE-SEM at an accelerating voltage of 15 kV and working distances of 8.7–8.9 mm. The scattering layer has large particles of hydrangea powder and small particles of P25-TiO_2_ powder.

**Figure 3:**
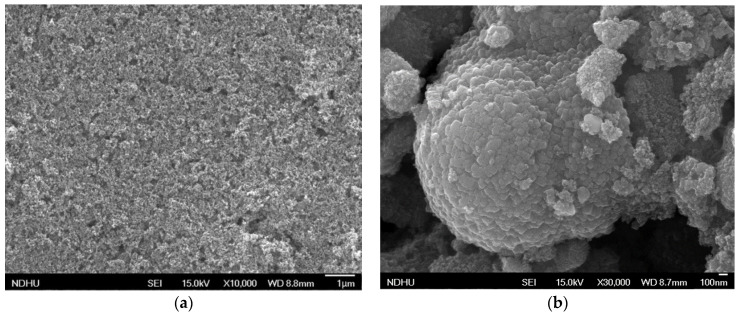
FE-SEM images showing the surface morphology of (**a**) P25 TiO_2_ and (**b**) H-TiO_2_ films.

Figure 3a displays the surface morphology of the P25 TiO_2_ film. The magnification is 10,000. The P25-TiO_2_ particles have an average size of around 20 nm. These particles are small and, therefore, have a high specific surface area. Figure 3b shows the surface morphology of the hydrothermally synthesized TiO_2_ film. The magnification is 30,000. The degree of aggregation is high, favoring the scattering of light.

**Figure 4:**
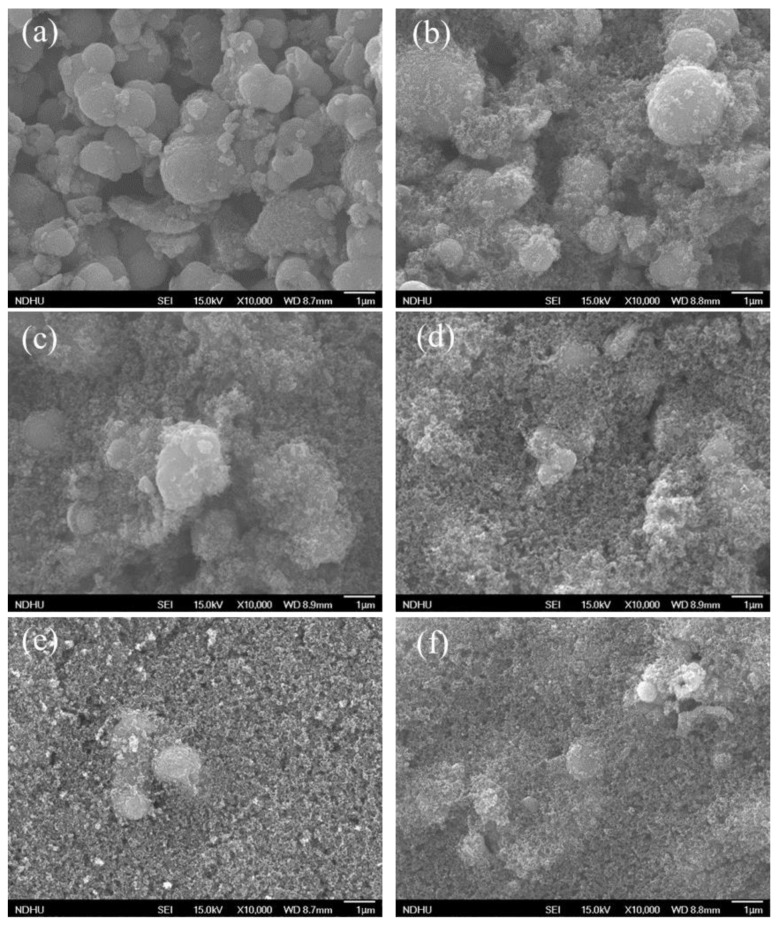
FE-SEM images of TiO_2_ with different proportions between H-TiO_2_ and P25 TiO_2_. (**a**) H-TiO_2_ only; (**b**) H-TiO_2_:P25 TiO_2_ = 9:1; (**c**) H-TiO_2_:P25 TiO_2_ = 7:3; (**d**) H-TiO_2_:P25 TiO_2_ = 5:5; (**e**) H-TiO_2_:P25 TiO_2_ = 3:7; (**f**) H-TiO_2_:P25 TiO_2_ = 1:9.

Figure 4 displays FE-SEM images of the scattering layer with different ratios of constituent materials, including H-TiO_2_ only. The size of H-TiO_2_ is about 300 nm to 2 μm. The magnification is 10,000. The figure clearly reveals that after adding P25 TiO_2_, the characteristics of the film are relatively dense. The small particles of P25 TiO_2_ increase surface area and dye adsorption capacity.

#### 3.1.3. Absorption Spectrum

The amount of dye that is absorbed by the TiO_2_ film is quantified by UV-vis spectroscopy desorption [11,18]. The absorption value at 515 nm was used to calculate the number of adsorbed N719 dye molecules, according to the Beer–Lambert law [12,35,37],

A_*dye*_ = ε_*dye*_
*l* C_*dye*_, (2)

where *A_dye_* is the absorbance of UV–visible light at a wavelength of 515 nm; *ε_dye_* is the molar extinction coefficient of the dye [10,12]; *l* is the path length of an optical cuvette (1 cm); and *C_dye_* is the molar dye concentration in the NaOH solution [10,12].

**Figure 5:**
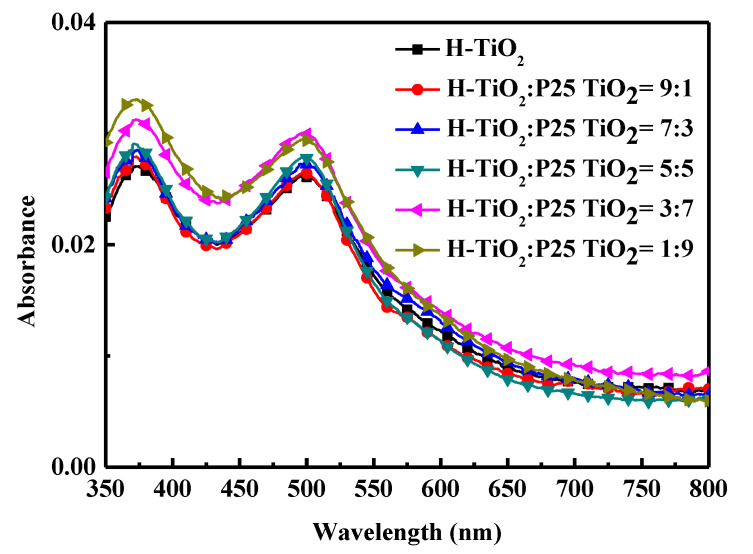
Absorption spectra of desorbed dye from the different scattering layers.

Figure 5 shows the absorbance of dye desorbed from the different scattering layers by NaOH. Table 3 lists the dye loading amount. Experiments indicate that the addition of small-particle P25 TiO_2_ increases the dye adsorption capacity. When the ratio is H-TiO_2_:P25 TiO_2_ = 3:7, the N719 dye loading capacity reaches its maximum value, which is 184.3 nmol/cm^2^. Accordingly, mixing H-TiO_2_ and P25 TiO_2_ in an appropriate ratio favors the adsorption of dye on the film, improving photovoltaic capacity.

### 3.2. Photovoltaic Characterization

#### 3.2.1. Analysis of DSSCs with 1-Layer P25 TiO_2_ and Single Scattering Layer

Screen printing is used to form single-layer P25 TiO_2_, and single-layer scattering layers are coated with different proportions of P25 TiO_2_ and H-TiO_2_. The 1-layer P25 TiO_2_ combined with a 1-layer H-TiO_2_ scattering layer [(H-TiO_2_): P25-TiO_2_ ratio = 10:0] is first studied to examine its potential effectiveness in DSSCs. Then, P25 TiO_2_ is mixed into the H-TiO_2_ scattering layer in various ratios to maximize the conversion efficiency.

**Figure 6:**
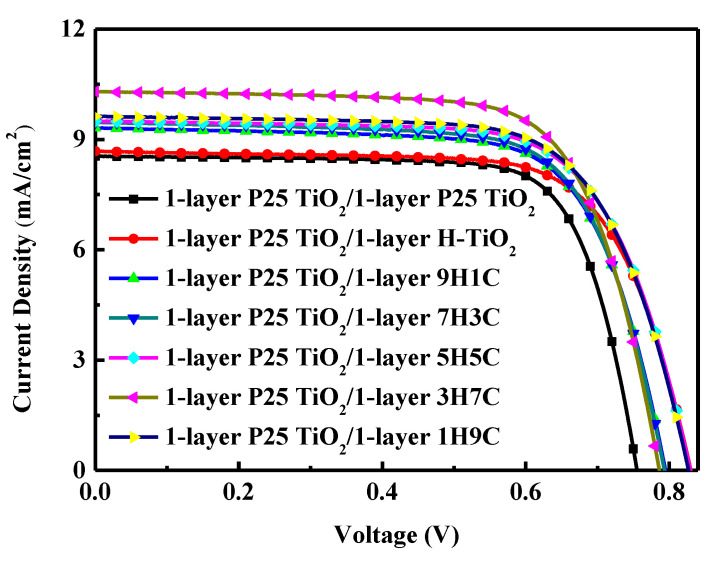
*J*-*V* characteristics of illuminated DSSCs with 1-layer P25 TiO_2_ and single scattering layer.

Photocurrent density-voltage (*J*-*V*) curves are plotted to characterize DSSCs directly under illumination. The photoelectrode has an area of 0.16 cm^2^. Figure 6 plots the *J*-*V* curves of DSSCs with 1-layer P25 TiO_2_ and different scattering layers. Table 4 provides the characteristic photovoltaic parameters of interest, which are obtained from Figure 6. Measurements are made of all solar cells under the same illumination conditions at an illumination level of 100 mW/cm^2^. Table 4 reveals that after the outermost P25 TiO_2_ layer is replaced with a H-TiO_2_ scattering layer, the short-circuit photocurrent and conversion efficiency are improved.

The solar energy-to-electricity conversion efficiency (*η*) of DSSC is expressed as [1,12,38].

(3) $$\eta =\frac{J_{sc}V_{oc}FF}{P_{in}}\times 100\%$$

where *J_sc_* is the short-circuit photocurrent density (mA/cm^2^); *V_oc_* is the open-circuit voltage (V); *P_in_* is the incident light power per unit area. The fill factor (FF) is expressed as [12,38]

(4) $$FF=\frac{P_{max}}{J_{sc}V_{oc}}=\frac{J_{max}V_{max}}{J_{sc}V_{oc}}$$

where *J_max_* and *V_max_* are the current and voltage, respectively, at the maximum power point in the *J-V* curves of the solar cells.

**Figure 7:**
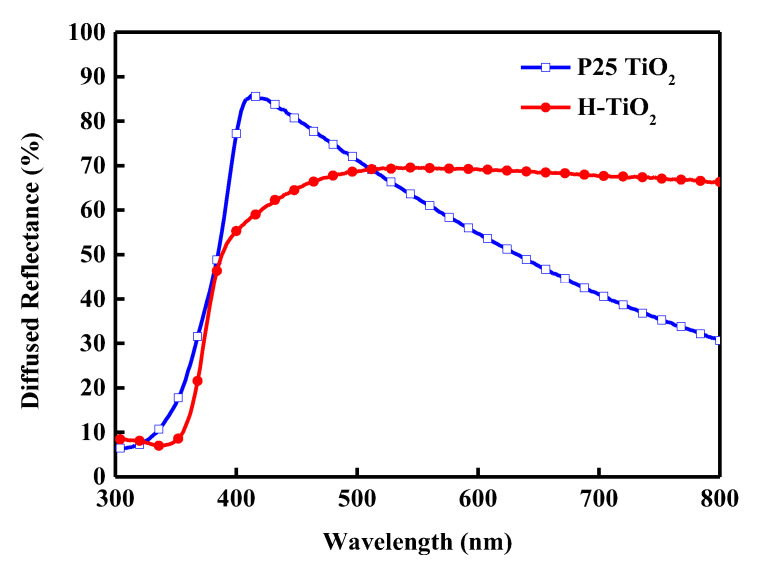
Diffused reflectance spectra of P25 TiO_2_ and H-TiO_2_ films without adsorbed dye.

To confirm the effects of the H-TiO_2_ scattering layer, reflectance was measured. Figure 7 displays the measured reflectance of P25 TiO_2_ and H-TiO_2_ films without adsorbed dye. The reflectance of P25 TiO_2_ gradually decreases in the band above 500 nm, indicating that the reflectance of P25 TiO_2_ becomes worse and the scattering becomes weaker. H-TiO_2_ retains a high reflectance at wavelengths above 500 nm, so it exhibits stronger overall scattering.

The above results prove that this scattering layer can indeed improve photovoltaic performance. In this experiment, when the (H-TiO_2_): P25 TiO_2_ ratio = 3:7, the DSSC generates the best short-circuit photocurrent and highest conversion efficiency. Increasing the ratio of P25 TiO_2_ not only increases the surface area but also increases the adsorption of dyes, improves photon scattering ability, and, thereby, increases the short-circuit current. However, if too much P25 TiO_2_ is added, then the scattering becomes weak because the proportion of hydrangea TiO_2_ is too small, so the short-circuit current is reduced. These results can be compared with those in Table 3. When the scattering layer ratio (H-TiO_2_):P25 TiO_2_ = 3:7, the device has the highest dye loading and J_SC_ of the studied structures.

#### 3.2.2. Analysis of DSSCs with 5-Layer P25 TiO_2_ and Single Scattering Layer

**Figure 8:**
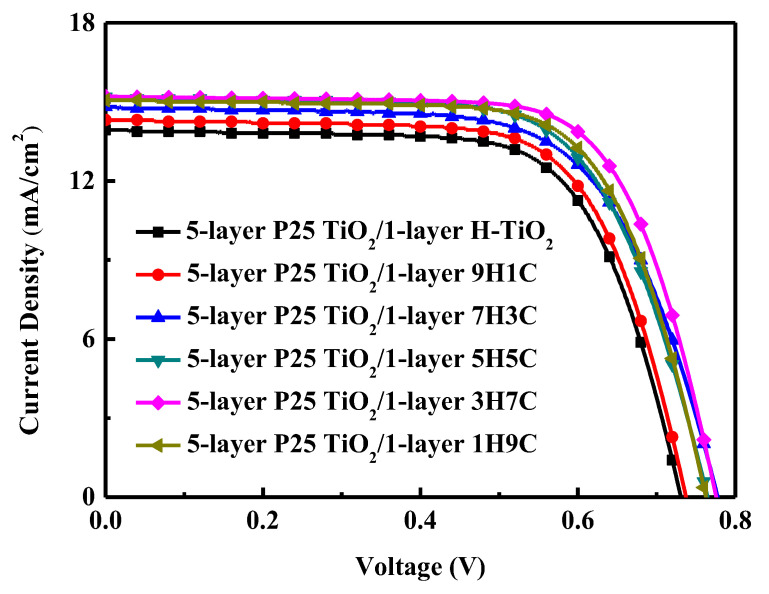
*J*-*V* characteristics of illuminated DSSCs with a 5-layer P25 TiO_2_ and single scattering layer.

In Section 3.2.1, the 1-layer P25 TiO_2_ DSSCs with different scattering layers are investigated. Experimental results demonstrate that the DSSC with the 3H7C scattering layer has the highest photoelectric conversion efficiency. Consequently, DSSCs with 5-layer P25 TiO_2_ and a single scattering layer with various parameters are also investigated to find the best (H-TiO_2_):P25 TiO_2_ ratio. Figure 8 plots the illuminated *J*-*V* curves, and Table 5 summarizes the photovoltaic characteristics. The thickness of the photoanode is about 25 mm. Coating the five-layer P25 TiO_2_ and single-layer 3H7C scattering layer yielded the highest current density of 15.26 mA/cm^2^, the largest open-circuit voltage of 0.77 V, the largest filling factor of 0.71, and the highest conversion efficiency of 8.33% of any of the studied devices. Measurements of more than three DSSCs of the same type were made. The variations in all DSSC characteristics were less than 3%. The ratio of P25 TiO_2_ and H-TiO_2_ is 3:7 is posited to maximize connectivity, resulting in no excessive fracture surfaces on the electrode surface and improved dye adsorption capacity.

Furthermore, IMVS and IMPS are used to evaluate the charge-collection efficiency (*η*_cc_) of the studied DSSCs. The recombination time (*τ_r_*) can be calculated from the equation $\tau_{r}=\frac{1}{2\pi f_{r}}$, where *f_r_* is the characteristic frequency minimum of the IMVS imaginary component. The collection (transport) time (*τ_c_*) can be calculated from the equation $\tau_{c}=\frac{1}{2\pi f_{c}}$, where *f_c_* is the characteristic frequency minimum of the IMPS imaginary component. *η*_cc_ is strongly determined by competition between charge collection and recombination. $\eta_{cc}=1-\frac{\tau_{c}}{\tau_{r}}$ [39,40]. Experimental results demonstrate that when H-TiO_2_ and P25-TiO_2_ are mixed, the electron transport time can be reduced. It proves that after mixing, its connectivity becomes better, which can make the electron transfer path smoother. The values of *τ_c_* and *τ_r_* for the DSSC with the 3H7C scattering layer are 7.98 ms and 200.36 ms, respectively. The DSSC with the 3H7C scattering layer has the shortest collection time, causing the largest charge-collection efficiency (*η*_cc_ = 0.96) of our studied DSSCs. This result is consistent with our *J-V* measurement in this work. Consequently, the DSSC with the 3H7C scattering layer has the largest *J*_sc_ and *η* of the studied DSSCs.

## 4. Conclusions

In this work, hydrangea-shaped TiO_2_ were hydrothermally synthesized and used in the scattering layer of DSSCs. Smaller particles of P25 TiO_2_ were mixed with larger H-TiO_2_ particles to form the scattering layer in the photoanode. When the (H-TiO_2_): P25 TiO_2_ ratio exceeded a particular value (3:7 in this study), as the H-TiO_2_ content increased, the short-circuit current and conversion efficiency decreased, mainly because the reduction in dye adsorption capacity results in a reduction of short-circuit current. Experimental results demonstrate that a suitable mixture of small and large particles in light-scattering layers enhances the conversion efficiency of DSSCs. In our future work, we will add graphene to the structure of the studied DSSC and investigate its impact on device characteristics.

## Figures and Tables

**Table 1:** Comparison of the photovoltaic characteristics of our proposed DSSC with those of the previously reported DSSCs.

Light Scattering Layer	*J*_sc_ (mA/cm^2^)	*V*_oc_ (V)	FF	*η* (%)	References
H-TiO_2_:P25 TiO_2_ = 3:7 (5-layer P25 TiO_2_)	15.26	0.77	0.71	8.33	This work
Coral-like TiO_2_	13.28	0.71	0.71	6.7	[15]
Hydrangea-likeTiO_2_	14.03	0.74	0.72	7.5	[15]
Hollow TiO_2_ nanoparticles (HTNPs)	16.26	0.68	0.72	8.08	[16]
TiO_2_ hierarchical micro-spheres and nanobelts	17.86	0.72	0.63	8.08	[17]
TiO_2_ hollow microspheres (THS) (1 wt%)	12.02	0.69	0.59	5.01	[18]
Anatase TiO_2_ nanowires with nanoscale whiskers	12.72	0.74	0.63	5.98	[19]
popcorn-like TiO_2_	13.95	0.77	0.7	7.56	[20]
worms-like TiO_⁠2_ nanostructures	14.77	0.77	0.62	7.05	[21]
TiO_2_ microspheres	13.32	0.78	0.63	6.49	[22]
TiO_2_ nanobelts	16.1	0.69	0.63	7.85	[23]
TiO_2_ nanoleaf	14.00	0.69	0.53	5.12	[24]
Nanofiber-structured TiO_2_	12.6	0.7	0.69	6.00	[25]
Flower-like TiO_2_	16.07	0.65	0.62	6.48	[26]
TiO_2_/graphene quantum dot (GQD)	14.22	0.69	0.51	5.01	[27]
50-nm ZnO/30-nm ZnO	18.99	0.67	0.46	5.87	[28]
TiO_2_/7.5% graphene	15.64	0.71	0.63	7.08	[29]

**Table 2:** Ratios of constituents of scattering paste with relevant notation.

Symbols of Scattering Paste	(H-TiO_2_):Commercial P25 TiO_2_ Ratio
9H1C	9:1
7H3C	7:3
5H5C	5:5
3H7C	3:7
1H9C	1:9

**Table 3:** Dye-loading of scattering layer film.

Sample	Dye Loading Amount(nmol/cm^2^)
H-TiO_2_	162.0
9H1C	162.3
7H3C	168.3
5H5C	169.5
3H7C	184.3
1H9C	182.5

**Table 4:** Photovoltaic properties of DSSCs with 1-layer working electrode and single scattering layer.

Photoanode	*J*_sc_ (mA/cm^2^)	*V*_oc_ (V)	FF	*η* (%)
1-layer P25/1-layer P25	8.54	0.76	0.74	4.82
1-layer P25/1-layer H-TiO_2_	8.68	0.83	0.71	5.09
1-layer P25/1-layer 9H1C	9.31	0.80	0.7	5.22
1-layer P25/1-layer 7H3C	9.47	0.79	0.71	5.29
1-layer P25/1-layer 5H5C	9.49	0.83	0.69	5.46
1-layer P25/1-layer 3H7C	9.93	0.77	0.73	5.58
1-layer P25/1-layer 1H9C	9.63	0.83	0.69	5.51

**Table 5:** Photovoltaic characteristics of DSSCs with a 5-layer working electrode and single scattering layer.

Photoanode	*J*_sc_ (mA/cm^2^)	*V*_oc_ (V)	FF	*η* (%)
5-layer P25/1-layer H-TiO_2_	13.94	0.74	0.68	7.03
5-layer P25/1-layer 9H1C	14.31	0.74	0.69	7.29
5-layer P25/1-layer 7H3C	14.81	0.76	0.68	7.62
5-layer P25/1-layer 5H5C	15.13	0.76	0.68	7.84
5-layer P25/1-layer 3H7C	15.26	0.77	0.71	8.33
5-layer P25/1-layer 1H9C	15.06	0.76	0.7	7.99

## Data Availability

The original contributions presented in this study are included in the article. Further inquiries can be directed to the corresponding author.
